# Anthropometry and Dietary Intake before and during a Competition in Mountain Runners

**DOI:** 10.1155/2014/893090

**Published:** 2014-08-07

**Authors:** Anja Carlsohn, Wolfram Müller

**Affiliations:** ^1^University of Education Schwäbisch Gmünd, Institute of Health Sciences, Oberbettringer Straße, 200 73525 Schwäbisch Gmünd, Germany; ^2^Medical University of Graz, Institute of Biophysics, Harrachgasse 21, 8010 Graz, Austria

## Abstract

Mountain running is a non-Olympic sport consisting of uphill or up- and downhill races at moderate-to-high altitude. Special nutritional requirements are anticipated, but no nutritional data of mountain runners are available. In three studies, physique of elite and recreational athletes (*N* = 62), maximum oxygen uptake (*N* = 3), and prerace and race day dietary intake (*N* = 6) were measured (mean ± SD). Mean oxygen uptake was 68.7 ± 5.2 mL/kg/min. Energy and carbohydrate intake before a race (29 ± 15 km, 1596 ± 556 m HD) was 3199 ± 701 kcal/d (13.4 ± 2.9 MJ/d) and 497 ± 128 g/d (8.3 ± 1.8 g/kg/d) in German national team members. Fluid intake was calculated as 2783 ± 1543 mL/d. During the race, athletes consumed 336 ± 364 kcal and 927 ± 705 mL of fluids. Substrate intake per hour was calculated as 23 ± 22 g of carbohydrates and 4.0 ± 3.2 g of proteins. In conclusion, anthropometric and oxygen uptake characteristics of mountain runners were similar to those reported for elite distance runners. Carbohydrate intake before and during the race was below recommendations for endurance athletes. This is of concern when considering the increased reliance on carbohydrates at altitude.

## 1. Introduction

Mountain running is a non-Olympic track and field sport. Under the auspices of the International Association of Athletics Federation (IAAF) and the World Mountain Running Association (WMRA, association under IAAF patronage), international championships in Africa, Europe, North America, Central America, and South America are conducted.

There are two main types of mountain running competitions: uphill races and up- and downhill events. Although the racetracks are of natural origin and are not artificially constructed, there are some course requirements stated by the WMRA. The length of the uphill tracks should approximate 8 km with an ascent of 800 m for females or 12 km with an ascent of 1200 m for males (10% ascent in average for both genders). The up- and downhill courses approximate a length of 8 km for females and 12 km for males, with ascents/descents of 500 m and 750 m, respectively.

Uphill races often finish at or above an altitude of 2500 m above sea level (a.s.l.), whereas up- and downhill courses most often start and finish at the same point at altitudes of 500 m a.s.l. or higher. Due to the shifting running conditions (surface, elevation, and direction) and increase in altitude, average running velocity in mountain races is much slower than during flat races. As a raw estimation, running time during a mountain race is 50% to 75% longer compared to the expected running time during flat races of the same distance. Drinking or eating is almost impossible at steep or rocky passages, where athletes breathe heavily and/or full concentration is required. According to the WMRA technical regulations, water and other “suitable” refreshments shall be made available at the finish and at intervals of 2-3 kilometres. Converted into running time, refreshment stations are available approximately every 10–30 minutes.

Mountain runners do not necessarily live and train in alpine landscapes all the year but divide their competition preparation into training periods at sea level and others at moderate or high altitude (3-4 weeks at 1800–3000 m a.s.l. for several times a year). Elite mountain runners conduct 10–14 training sessions per week and cover a weekly running distance of 120–200 km. During altitude training camps, up to twenty training sessions per week are conducted with a focus on repeated uphill runs and long-distance sessions with a cumulative gain in altitude of 500–1000 m (personal communication to German national team members).

The unique physiological strain on mountain runners during training and competition suggests special nutritional demands. However, little is known about the nutrition and physique of mountain runners. The purpose was to collect a set of data (1) on the physique of mountain runners and (2) on the dietary intake at the day before and during mountain running competitions of elite and recreational mountain runners and to compare the results from this sample to nutritional habits in other endurance sports. We hypothesized that elite mountain runners have a low body mass, as this might be a competitive advantage. We also hypothesized that the dietary intake during the race does not meet the recommendations for endurance runners, as the nutritional supply of the athletes might be difficult in the mountains.

## 2. Materials and Methods

Three independent pilot studies were conducted to collect preliminary data about physique and endurance capacity, prerace, and race day dietary intake of mountain runners. All volunteers gave written informed consent before participating in the studies. The assessment of anthropometric characteristics, dietary intake, and maximum oxygen uptake of athletes was approved by the Ethical Committee of the University of Potsdam. The studies were carried out according to the Declaration of Helsinki.

### 2.1. Anthropometry

The day before an international mountain running competition that served as qualification race for the European Championships (Feuerkogel Mountain Race, Austria: length 10 km, 1250 gain in altitude), anthropometric data of 62 elite and recreational athletes were collected. Here, age, body mass, and body height were measured with the barefoot subjects standing in an upright position [1]. Dressed body mass was corrected by 1 kg to account for the clothes worn by the athletes, and body-mass index (BMI) was calculated using the corrected body mass and the body height.

### 2.2. Maximum Oxygen Uptake

Maximum oxygen uptake ($\dot{V}{\text{O}}_{2}$max) as a surrogate parameter of endurance capacity was measured in three elite athletes from the German national team (2 females, 1 male). Here, a stepwise incremental running test on a treadmill was conducted with a starting velocity of 6 km/h for females and 8 km/h for the male athlete and an increment of 2 km/h every 3 minutes until exhaustion as described elsewhere [2]. Oxygen uptake and carbon dioxide expiration were measured continuously using a face mask with a stationary breath-by-breath system (ZAN 600 metabolic device, nSpire, Health, Oberthulba, Germany).

### 2.3. Prerace and Race Day Nutrition

Dietary intake was measured at the day before a mountain running competition in six elite mountain runners of the German national team (5 males, 1 female) using a 24-hour diet recall. Athletes were asked to collect their nutritional data before and during their upcoming competitions. Thus, data were collected at four different competitions: Dachstein Mountain race (12,1 km with 884 m gain in altitude, Dachstein, Austria), Inferno Half Marathon (21,1 km with 2175 m gain in altitude, Lauterbrunnen, Switzerland), LGT Alpin Marathon (42,2 km with 1870 m gain altitude, Malbun, Liechtenstein), and finally Schauinsland Mountain race (13 km with 905 m gain in altitude, Freiburg, Germany). In all four races, a regular supply approximately every 5 km was ensured.

To analyze the dietary intake during the race, athletes were asked to complete a diet recall at the same day for the race only. Athletes were instructed to use household measures (i.e., cups, packages, etc.) to record the amounts of food and fluids consumed. Energy and macronutrient intake was analysed based on the German food database BLS II.3 using PRODI expert software (PRODI NutriScience, Hausach, Germany). For foods not contained in the database such as energy bars or carbohydrate gels, standardized recipes according to the company's food labeling were created. For items not listed in the food database as well as for composite dishes a coding list was established to standardize the data input. To avoid interexaminer set-offs when transferring the food records into nutrient calculation [3], data input into the database was performed by one examiner only. All data are given as means ± standard deviations and 95% confidence intervals (CI). Student's *t*-test was conducted to test for gender differences where appropriate (alpha = 0.05).

## 3. Results

### 3.1. Age and Anthropometry

Mean age of elite and recreational participants of a qualification race for the European Championships was 37.3 ± 11.5 years (95% CI: 34.1–40.5 years). Body height was 1.76 ± 0.07 m (95% CI: 1.74–1.78 m), and body weight was measured as 65.3 ± 8.8 kg (95% CI: 63.0–67.5 kg). BMI was calculated as 21.0 ± 1.8 kg/m² (95% CI: 20.5–21.4 kg/m²), with significantly higher values in males than females (21.4 ± 1.7 kg/m² versus 19.6 ± 1.7 kg/m², *P* = .009). Anthropometric data for males and females are detailed in Table 1.

### 3.2. Maximum Oxygen Uptake

The $\dot{V}{\text{O}}_{2}$max measured in three members of the German national mountain running team was 64 and 66 mL/kg/min for the two females and 76 mL/kg/min for the male athlete.

### 3.3. Prerace and Race Day Nutrition

The distance covered during the mountain running competitions, where prerace and race diets were observed, was 28.8 ± 15.0 km with a gain in altitude of 1596 ± 556 m. Running time was 2 hours 25 minutes ± 1 hour 7 minutes. Total energy intake at the day before competition was 3199 ± 701 kcal/d (95% CI: 2463–3935 kcal/d). At the prerace day, athletes ingested 497 ± 128 g/d of carbohydrates (95% CI: 362–632 g/d), which corresponds to 8.3 ± 1.8 g/kg body mass/d (95% CI: 6.4–10.2 g/kg/d, Table 2). A protein intake of 99.9 ± 29.2 g/d of protein (95% CI: 69.1–130.4 g/d) was documented, which corresponds to 1.6 ± 0.3 g/kg body mass/d (95% CI: 1.3–2.0 g/kg/d). Fat intake was calculated as 85.8 ± 30.8 g/d (95% CI: 53.6–118.1 g/d). Total fluid intake was 2783 ± 1409 mL/d at the day before competition, with a 95% CI of 1164–4403 mL/d.

During the race, athletes preferred consuming liquid energy sources such as sports drinks, caffeine containing soft drinks, and/or commercial carbohydrate gels. Altogether, athletes ingested 336 ± 364 kcal while running (95% CI: −46–717 kcal). Mean carbohydrate intake was 68 ± 76 g, which corresponds to 23 ± 22 g carbohydrate per hour competition (95% CI: 0–46 g/h carbohydrates). Total fluid intake during the race was calculated 927 ± 704 mL/race (449 ± 376 mL per hour competition). Athletes consumed 4.0 ± 3.2 g of protein per hour (95% CI: 0.7–7.3 g/h) and 0.3 ± 0.5 g of fat per hour competition (95% CI: −0.2–0.8 g/h).

## 4. Discussion 

Considering the few available data [4, 5], elite mountain runners seem to have similar anthropometric and physiological characteristics compared to distance runners. In participants of a WMRA Masters World Championship (46 ± 9 years) a body mass of 64.5 ± 7.9 kg and a height of 177 ± 8 cm were measured [4], which is comparable to elite middle-distance runners [6]. In most endurance sports, low body fat is of advantage, as this improves the power-to-weight ratio [7]. Mountain running athletes also had a low body mass which indicates that low body weight may increase running efficiency, at least as long as it is above critically low body weight, which may decrease $\dot{V}{\text{O}}_{2}$max relative to body mass and increase running time [8]. In an experimental setting, an additional 7.5% of weight resulted in a 30% reduction of running performance in male runners [9, 10].

Furthermore, energy cost for running was shown to considerably increase with steepness on both ascents and descents compared to running or walking at level [11]. In a single case analysis, a remarkable increase in energy expenditure was observed during uphill walking when either weight, speed, slope, or altitude was augmented [12]. Thus, a low body mass may be of special importance for mountain runners who compete on steep trails at altitude. This issue might explain the relatively low body mass in both male and female runners. However, as body mass or body mass index is of limited value for the use in athletes, information regarding body composition of mountain runners is required for interpreting data on a finer scale.

Regarding aerobic capacity, $\dot{V}{\text{O}}_{2}$max measured in three internationally elite mountain runners was quite similar to values observed in master mountain runners with a $\dot{V}{\text{O}}_{2}$max of 67.8 ± 6.9 mL/kg/min [4]. The values of $\dot{V}{\text{O}}_{2}$max measured in the current study in elite mountain runners are close to results reported for elite marathoners. Typically, elite marathon runners achieve values of 70 mL/kg/min for males and 60 mL/kg/min for females, respectively [13].

The dietary intake at the day before competition might be considered generally adequate, although the interindividual variation in energy, carbohydrate, and fluid intake was high.

The mean observed carbohydrate intake of 8.5 g/kg/d at the day before competition is lower than recommended to optimize glycogen stores before a marathon [14]. However, as no information is available about the nutrition and tapering strategies of the mountain runners during the last 3–5 days before the competition, conclusions about the adequacy of carbohydrate intake in mountain runners before the race cannot be drawn.

Carbohydrate utilization during exercise and postexercise recovery is higher in hypoxia than at sea level, increasing the athletes' reliance on carbohydrate-rich foods [15]. This is a special challenge for endurance athletes with high training volumes at moderate-to-high intensities, as the important fuel source glycogen rapidly becomes a limiting factor. However, this effect is more pronounced in men than in women, who utilize fat to a greater extent at altitude than men [16]. In addition to a relatively low carbohydrate intake before the race, carbohydrate consumption of elite mountain runners during the race was found to be lower than recommended. Currently, for endurance events a carbohydrate intake of 30–60 g/hour is recommended [17], and there is growing evidence for beneficial effects of even higher dosages during competitions lasting longer than 2.5 hours (for review, see [18]).

However, due to the increased carbohydrate reliance at altitude, mountain runners competing at moderate to high altitudes may require even more carbohydrates than athletes competing at sea level. At very high altitude (>5000 m a.s.l.), carbohydrate supplementation was shown to improve time trial performance by 17% and reduce ratings of perceived exertion by 18% [19]. Thus, increasing carbohydrate ingestion during the race might be recommended to the athletes.

Interestingly, elite mountain runners consumed relatively large amounts of protein at the prerace day, pretty close to what is recommended for endurance athletes during heavy training periods. This observation is found consistently in all six elite mountain runners, although nutritionists might recommend reducing the protein intake in favor of carbohydrates.

Water intake during the race was highly variable. As no data are available on sweat rates and water loss during mountain running in the altitude, it remains elusive whether the ingested amount of ~400 mL per hour is sufficient to prevent dehydration higher than 2% of body mass.

To our knowledge, this is the first time that data on anthropometry, $\dot{V}{\text{O}}_{2}$max, and prerace and race day nutrition in WMRA mountain runners are presented. However, the study has some limitations that need to be considered when interpreting the data. Firstly, the data were collected in three different pilot studies, which means that anthropometric, nutritional, and $\dot{V}{\text{O}}_{2}$max data were in part collected in different subjects. Secondly, the sample sizes in the two pilot studies with elite athletes only ($\dot{V}{\text{O}}_{2}$max measurements and nutritional data) are very limited with three and six athletes and represent a case series rather than a study. Therefore, this first set of data presented here is preliminary and should be extended in further studies including larger number of subjects and coherent data sets in all subgroups. However, it is difficult to motivate international elite athletes to participate in studies, where data are collected shortly before or even during the race. In addition, the number of mountain runners is much smaller than in Olympic sports or in sports that are commonly exerted in the general population such as cycling.

Thirdly, there are some issues about the data collection of the race day nutrition. Here, a recall for the race time only was used, where athletes had to record their estimated intake as soon as possible after the race. A recall of the dietary intake during the race might be biased by the remembrance of the individuals.

## 5. Conclusions 

In conclusion, little data are available on nutrition and anthropometry of elite mountain runners. Preliminary date of this study shows that carbohydrate intake of mountain runners before and during the race is low when compared to distance runners. Therefore, increase of carbohydrate intake may increase performance in mountain running, but this can only be hypothesized on the basis of data presented here. Energy intake was rather low, which might reflect the challenge to find a balance between low body mass and adequately fueled glycogen stores. More studies are needed to create reliable nutrition recommendations in terms of performance optimization for elite mountain runners.

## Figures and Tables

**Table 1:** Anthropometric characteristics of elite and recreational mountain runners participating at a qualifying for the European Championships.

	General cohort (*N* = 62)	Females (*N* = 14)	Males (*N* = 48)
Age (years)	37.3 ± 11.5	35.8 ± 9.3	37.7 ± 12.1
Height (cm)	176 ± 7	167 ± 4^a^	178 ± 6^a^
Body mass (kg)	65.3 ± 9.8	54.8 ± 6.09^b^	68.4 ± 6.9^b^
BMI (kg/m^*2*^)	21.0 ± 1.8	19.6 ± 1.7^c^	21.4 ± 1.7^c^

Data are given as means ± SD.

a, b, c indicate significant differences between the genders (*P* > 0.05).

**Table 2:** Prerace day nutrition and dietary intake during mountain races in *N* = 6 elite athletes.

	Prerace day diet	Race diet
Energy intake	3199 ± 701 kcal/day (2463; 3935)	336 ± 364 kcal/race (−46; 717)
Fluid intake	2783 ± 1543 mL/day (1164; 4403)	927 ± 705 mL/race (187; 1665)
Relative water intake	—	449 ± 376 mL/hour (55; 844)
Carbohydrate intake	497 ± 128 g/d (362; 632)	68 ± 76 g/race (−11; 148)
Relative carbohydrate intake	8.3 ± 1.8 g/kg/d (6.4; 10.2)	23 ± 22 g/hour (0; 46)
Protein intake	99.8 ± 29.2 g/d (69; 130)	11 ± 11 g/race (0; 22)
Relative protein intake	1.65 ± 0.31 g/kg/d (1.32; 1.97)	4.0 ± 3.2 g/hour (0; 46)
Fat intake	85.8 ± 30.8 g/d (53.6; 118.1)	0.7 ± 1.6 g/race (−1.0; 2.4)
Relative fat intake	1.4 ± 0.5 g/kg/d (0.85; 1.95)	0.3 ± 0.5 g/hour (−0.2; 0.8)

Data are given as mean ± SD (upper; lower 95% CI).

## References

[1] Ackland TR, Lohman TG, Sundgot-Borgen J (2012). Current status of body composition assessment in sport: review and position statement on behalf of the Ad Hoc research working group on body composition health and performance, under the auspices of the I.O.C. medical commission. *Sports Medicine*.

[2] Scharhag-Rosenberger F, Carlsohn A, Cassel M, Mayer F, Scharhag J (2011). How to test maximal oxygen uptake: a study on timing and testing procedure of a supramaximal verification test. *Applied Physiology, Nutrition and Metabolism*.

[3] Braakhuis AJ, Meredith K, Cox GR, Hopkins WG, Burke LM (2003). Variability in estimation of self-reported dietary intake data from elite athletes resulting from coding by different sports dietitians. *International Journal of Sport Nutrition and Exercise Metabolism*.

[4] Burtscher M, Förster H, Burtscher J (2008). Superior endurance performance in aging mountain runners. *Gerontology*.

[5] Knechtle B, Knechtle P, Rosemann T (2010). Race performance in male mountain ultra-marathoners: anthropometry or training?. *Perceptual and Motor Skills*.

[6] Arrese AL, Ostáriz ES (2006). Skinfold thicknesses associated with distance running performance in highly trained runners. *Journal of Sports Sciences*.

[7] O'Connor H, Olds T, Maughan RJ (2007). Physique and performance for track and field events. *Journal of Sports Sciences*.

[8] Cureton KJ, Sparling PB, Evans BW, Johnson SM, Kong UD, Purvis JW (1978). Effect of experimental alterations in excess weight on aerobic capacity and distance running performance. *Medicine and Science in Sports and Exercise*.

[9] Cureton KJ, Sparling PB (1980). Distance running performance and metabolic responses to running in men and women with excess weight experimentally equated. *Medicine and Science in Sports and Exercise*.

[10] Malina RM (1975). Anthropometric correlates of strength and motor performance. *Exercise and Sport Sciences Reviews*.

[11] Minetti AE, Moia C, Roi GS, Susta D, Ferretti G (2002). Energy cost of walking and running at extreme uphill and downhill slopes. *Journal of Applied Physiology*.

[12] Koehler K, Huelsemann F, De Marees M, Braunstein B, Braun H, Schaenzer W (2011). Case study: simulated and real-life energy expenditure during a 3-week expedition. *International Journal of Sport Nutrition and Exercise Metabolism*.

[13] Davies CTM, Thompson MW (1979). Aerobic performance of female marathon and male ultramarathon athletes. *European Journal of Applied Physiology and Occupational Physiology*.

[14] Burke LM (2007). Nutrition strategies for the marathon: fuel for training and racing. *Sports Medicine*.

[15] Katayama K, Goto K, Ishida K, Ogita F (2010). Substrate utilization during exercise and recovery at moderate altitude. *Metabolism*.

[16] Braun B, Butterfield GE, Dominick SB (1998). Women at altitude: changes in carbohydrate metabolism at 4,300-m elevation and across the menstrual cycle. *Journal of Applied Physiology*.

[17] Rodriguez NR, DiMarco NM, Langley S (2009). Position of the American dietetic association, dietitians of Canada, and the american college of sports medicine: nutrition and athletic performance. *Journal of the American Dietetic Association*.

[18] Burke LM, Hawley JA, Wong SHS, Jeukendrup AE (2011). Carbohydrates for training and competition. *Journal of Sports Sciences*.

[19] Oliver SJ, Golja P, MacDonald JH (2012). Carbohydrate supplementation and exercise performance at high altitude: a randomized controlled trial. *High Altitude Medicine and Biology*.

